# Rescue Protocols for Canine Non‐Indolent B Cell Lymphoma: A Systematic Review

**DOI:** 10.1111/vco.70048

**Published:** 2026-02-08

**Authors:** Claire Marie Cannon, Peter Bennett

**Affiliations:** ^1^ Melbourne Veterinary School, Faculty of Science The University of Melbourne Parkville Victoria Australia

**Keywords:** dogs, drug therapy, lymphoma

## Abstract

Canine lymphoma is a heterogenous group of diseases. The most common subtype is diffuse large B cell lymphoma (DLBCL), though definitive diagnosis beyond non‐indolent = multicentric B cell lymphoma is not often achieved in clinical practice as it requires histopathology. Most dogs respond well to standard‐of‐care multiagent chemotherapy (CHOP) but relapse and eventual CHOP‐resistance is likely. Less commonly there is a lack of complete response to initial CHOP treatment. CHOP‐resistant cases are treated with rescue chemotherapy protocols, of which many are published, but the most effective is unknown. In this systematic review we aimed to determine the most effective rescue chemotherapy protocol for dogs with multicentric non‐indolent B cell lymphoma resistant to initial chemotherapy with a CHOP‐based protocol. After initial screening, 65 full‐text articles were reviewed. However, outcomes for the population of interest could not be identified in any, leaving our research question unanswered. Future publications of rescue treatment for canine lymphoma should report outcomes separately for groups of dogs where disease characteristics and prior treatment may affect outcome.

## 1 Introduction

Lymphoma in domestic dogs is a heterogeneous group of diseases with different clinical presentations and prognoses. The most common presentation is multicentric, with involvement of multiple lymph nodes and frequently other organs including liver, spleen, blood, and bone marrow. Within the category of multicentric lymphoma, there are multiple subtypes, diffuse large B cell lymphoma (DLBCL) being the most common [1].

The current standard of care treatment for DLBCL in dogs is combination chemotherapy, which includes cyclophosphamide, doxorubicin, vincristine, and prednisone/prednisolone (CHOP). Most dogs with multicentric lymphoma in general, and DLBCL specifically, respond well to CHOP but eventually relapse [2]. Re‐induction with CHOP is often effective in dogs that relapse after achieving complete remission [3], but eventually resistance to CHOP is expected to develop. In the published literature, up to approximately 4% of dogs with DLBCL fail to respond to initial CHOP chemotherapy [2, 4]. In cases where lymphoma is resistant to CHOP, either during initial induction or after relapse, so‐called ‘rescue’ chemotherapy protocols are often attempted.

Many rescue protocols for treatment of canine lymphoma have been reported and previously summarised [5] and it appears that responses are common but often short‐lived (weeks to months) [5]. However, the populations included in these previous reports are heterogeneous regarding lymphoma subtype, disease status (i.e., relapsed post CHOP completion compared with progressive disease or lack of response during CHOP treatment), and the number of rescue protocols dogs have received.

The aim of this systematic review was to examine the published literature concerning rescue protocols for canine lymphoma to attempt to answer the question:

In dogs with multicentric non‐indolent B cell lymphoma resistant to initial chemotherapy with a CHOP‐based protocol, what is the most effective first rescue protocol?

## 2 Methods

Criteria for inclusion of records in the systematic review were: original, peer‐reviewed retrospective or prospective studies, available in English, in which the population of interest could be identified, that is, dogs with multicentric non‐indolent (non‐small cell, non‐low grade, or known non‐indolent subtype) B cell lymphoma resistant to CHOP, treated with a first rescue protocol. ‘Resistant’ was defined as lack of complete response (CR) during the initial CHOP protocol, or progressive disease during CHOP or within 30 days of completion of CHOP. Studies could include dogs with other types of lymphoma, if outcomes for the population of interest could be identified separately. Outcome reporting was required, which could include response rates, remission duration, progression free interval, progression free survival, or overall survival time post rescue. Studies were excluded if they contained only dogs with other forms of lymphoma, dogs treated with rescue chemotherapy but not considered resistant to CHOP, dogs treated with second or subsequent rescue protocols, or studies where the outcomes for the population of interest could not be evaluated separately. Date and journal of publication were not selection criteria.

Online literature databases (PubMed and Web of Science) were searched between 19 November and 21 November 2024 to identify records. Following the literature search, additional records were identified via explicit manual searching using references in studies included in the full‐text review and review of related articles in PubMed.

PubMed was searched for: ‘canine’ OR ‘dog’ AND lymphoma AND relapse AND treatment, ‘canine’ OR ‘dog’ AND lymphoma AND rescue AND treatment, ‘canine’ OR ‘dog’ AND lymphoma AND resistant AND treatment, ‘canine’ OR ‘dog’ AND lymphoma AND refractory AND treatment. Web of Science was searched for: ‘canine’ AND ‘resistant lymphoma’ AND treatment, ‘canine’ AND ‘refractory lymphoma’ AND treatment. These multiple searches were performed to try to retrieve all potentially relevant records, given the lack of consistent use of terminology in the veterinary literature regarding relapsed, resistant, and refractory lymphoma and the meaning of rescue chemotherapy.

One investigator (CC) performed the database searches and initial review. Initial review consisted of two phases—title and abstract screening and full‐text screening. Based on screening, studies selected for systematic review would have data extracted by both investigators independently into a spreadsheet. Data extracted from each record in systematic review would include study characteristics (retrospective or prospective, single or multicentre), geographic location, number of dogs in the population of interest, rescue chemotherapy protocol evaluated, use of concurrent corticosteroids, response assessment criteria, overall response rates, other outcomes (progression free interval, remission duration, progression free survival, overall survival post rescue), and any prognostic factors for outcome identified in the study. The key outcomes of interest to be compared between rescue chemotherapy protocols were overall response rates and progression free interval.

Risk of bias was assessed at the study level based on the study design, including retrospective versus prospective, inclusion and exclusion criteria, response criteria, follow up and outcome assessment.

## 3 Results

**FIGURE 1:**
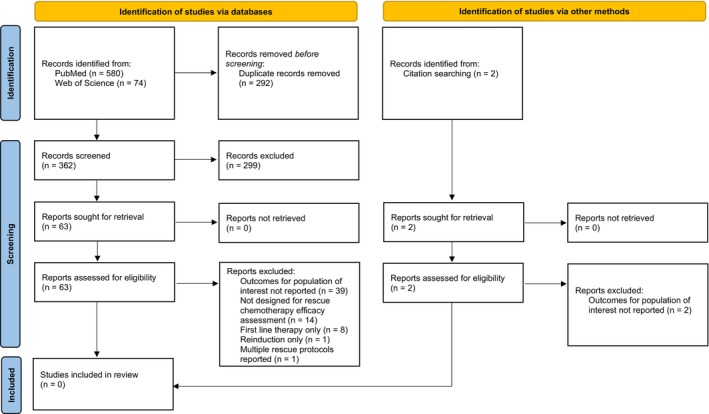
PRISMA flow diagram [6] for identification and screening of records.

A total of 654 records were retrieved from database searches. An additional two records were identified through references from screened articles. A total of 292 duplicate records were excluded; 299 were excluded based on the screening of the title or the abstract. Sixty‐five full‐text records (see Appendix 1) were reviewed and all were excluded. No articles met the criteria for inclusion in the systematic review (Figure 1).

## 4 Discussion

Despite many publications describing outcomes in dogs with lymphoma treated with rescue chemotherapy, our research question is unanswered. The populations of dogs included in the publications to date are not uniform, including dogs with different subtypes of lymphoma, dogs with relapsed disease that were not re‐induced with CHOP, and dogs with CHOP‐resistant lymphoma included together, and dogs with varying previous treatments, including differing induction protocols and variable numbers of rescue protocols. Most studies report results for the whole study population together, hindering interpretation. Even in the most recent studies [7, 8] including only dogs with non‐indolent multicentric lymphoma, where dogs with CHOP‐resistant lymphoma treated with first rescue chemotherapy protocols can be identified, immunophenotyping is incomplete and results are not reported separately for dogs with B and T cell lymphoma.

The population of interest for this review was selected as the most common population treated with chemotherapy for lymphoma, and therefore the most common to require rescue chemotherapy. Diagnosis of DLBCL compared with other non‐indolent B cell nodal lymphomas in dogs requires histopathology, since different B cell subtypes cannot reliably be distinguished based on other diagnostic tests such as flow cytometry, immunocytochemistry, and PCR clonality testing. Since differences in treatment response have not been shown amongst different B cell subtypes, it was deemed appropriate to consider these together. Indeed, the definition of ‘indolent’ B cell lymphoma is controversial, since subtypes considered ‘indolent’ can still have aggressive clinical behaviour [9], and so it could be argued that including indolent and non‐indolent multicentric B cell lymphomas may be appropriate for this review. Even if our inclusion criteria were expanded, it would still be impossible to identify outcomes for these dogs separately in the available literature.

Dogs with lymphoma resistant to initial CHOP treatment were selected as the most appropriate population for evaluation of rescue protocol efficacy as this is likely to represent a more homogenous population than dogs who relapse at varying time points post CHOP and are reinduced one or more times before being deemed CHOP‐resistant. There is no standardised definition of ‘resistant’ lymphoma in veterinary medicine, but relapse within 30 days post treatment of CHOP was defined as resistant in this study as has been done in others [7]. Again, even if our inclusion criteria were relaxed, there is no way to identify the population of interest in the current literature.

Outcomes for most studies screened here have been summarised previously in a textbook [5], with outcomes tabulated and appropriate commentary that comparison is inappropriate given the heterogeneity of the populations and risk of bias. There were three more recent publications identified in this review, which have not previously been summarised but are subject to the same biases [7, 8, 10]. It was hoped that by close review of previously summarised and more recent literature, outcomes for a less biased and still clinically relevant population could be identified, enabling comparison of different protocols. However, this was not possible. It may be possible to undertake new analysis if raw data is obtainable from previously published studies, but it is likely that numbers will still be too small for robust statistical analysis.

There are many clinical endpoints that can be evaluated in oncology, and the most appropriate to assess the effectiveness of therapy depends on the context of the disease, population, and treatment [11]. The most appropriate endpoint to determine the effectiveness of treatment in dogs with CHOP‐resistant DLBCL, or indeed any other cancer type, is not defined. In this study, our inclusion criteria for endpoint reporting were broad, in order to capture as many articles as possible. However, differences may hamper comparisons between studies. Studies reviewed here report different endpoints including progression free survival [7, 12], remission duration or duration of response [13, 14], disease free interval [15], time to progression or progression free interval [16, 17] and time to discontinuation of rescue treatment [18]. We selected progression free interval along with overall response rate as key outcome measures to objectively assess the anti‐tumour effect of rescue chemotherapy. Progression free survival in this setting is clinically relevant given that most dogs will die of progressive disease and this will also capture dogs that die of treatment related complications. Future studies of rescue therapy in dogs with DLBCL should report at least progression free survival as this is the most common endpoint in more recent studies.

The most effective rescue protocol for dogs with CHOP‐resistant non‐indolent B cell lymphoma, or indeed any other type of lymphoma, cannot be determined from the existing literature. Future studies of any rescue therapy for canine lymphoma, whether a novel chemotherapy protocol, radiotherapy, targeted therapy, based on ex vivo drug sensitivity [19], or another approach, should use a clear definition of resistant disease and must report outcomes separately for dogs where disease characteristics are likely to impact the outcome—that is, based on lymphoma subtype (including immunophenotype) and prior treatment. Investigators must resist the impulse to bolster numbers by reporting outcomes for a heterogenous patient population together, as this only serves to render the data less useful.

## Funding

The authors have nothing to report.

## Conflicts of Interest

The authors declare no conflicts of interest.

## Supporting information


**Appendix 1** Full‐text articles screened for systematic review.

## Data Availability

The data that support the findings of this study are available from the corresponding author upon reasonable request.
