# Supplementary material for: Rescue Protocols for Canine Non‐Indolent B Cell Lymphoma: A Systematic Review
Source: Vet Comp Oncol. 2026 Feb 8;24(2):416–9. doi: 10.1111/vco.70048 (PMC13161734; doi:10.1111/vco.70048)
Supplement: Supplementary file 1 — Appendix 1 Full‐text articles screened for systematic review. [file VCO-24-416-s001.docx]

**Appendix 1**

Full text articles screened for systematic review

1. Alvarez FJ, Kisseberth WC, Gallant SL, Couto CG. Dexamethasone, Melphalan, Actinomycin D, Cytosine Arabinoside (DMAC) Protocol for Dogs with Relapsed Lymphoma. *Journal of Veterinary Internal Medicine*. 2006;20(5):1178-1183. doi:10.1111/j.1939-1676.2006.tb00719.x

2. Back AR, Schleis SE, Smrkovski OA, Lee J, Smith AN, Phillips JC. Mechlorethamine, vincristine, melphalan and prednisone ( MOMP ) for the treatment of relapsed lymphoma in dogs. *Veterinary and Comparative Oncology*. 2015;13(4):398-408. doi:10.1111/vco.12055

3. Bannink EO, Sauerbrey ML, Mullins MN, Hauptman JG, Obradovich JE. Actinomycin D as rescue therapy in dogs with relapsed or resistant lymphoma: 49 cases (1999–2006). *Journal of the American Veterinary Medical Association*. 2008;233(3):446-451. doi:10.2460/javma.233.3.446

4. Batschinski K, Dervisis N, Kitchell B, Newman R, Erfourth T. Combination of Bleomycin and Cytosine Arabinoside Chemotherapy for Relapsed Canine Lymphoma. *Journal of the American Animal Hospital Association*. 2018;54(3):150-155. doi:10.5326/JAAHA-MS-6617

5. Benjamin SE, Sorenmo KU, Krick EL, et al. Response-based modification of CHOP chemotherapy for canine B-cell lymphoma. *Veterinary and Comparative Oncology*. 2021;19(3):541-550. doi:10.1111/vco.12693

6. Blaxill JE, Bennett PF. Evaluation of clinical response and prognostic factors in canine multicentric lymphoma treated with first rescue therapy. *Veterinary and Comparative Oncology*. 2024;22(2):265-277. doi:10.1111/vco.12974

7. Brodsky EM, Maudlin GN, Lachowicz JL, Post GS. Asparaginase and MOPP Treatment of Dogs with Lymphoma. *Journal of Veterinary Internal Medicine*. 2009;23(3):578-584. doi:10.1111/j.1939-1676.2009.0289.x

8. Burton JH, Mazcko C, LeBlanc A, et al. NCI Comparative Oncology Program Testing of Non-Camptothecin Indenoisoquinoline Topoisomerase I Inhibitors in Naturally Occurring Canine Lymphoma. *Clinical Cancer Research*. 2018;24(23):5830-5840. doi:10.1158/1078-0432.CCR-18-1498

9. Callegari AJ, Tsang J, Park S, et al. Multimodal machine learning models identify chemotherapy drugs with prospective clinical efficacy in dogs with relapsed B-cell lymphoma. *Frontiers in Oncoogyl*. 2024;14:1304144. doi:10.3389/fonc.2024.1304144

10. Calvert CA, Leifer CE. Doxorubicin for treatment of canine lymphosarcoma after development of resistance to combination chemotherapy. *Journal of the American Veterinary Medical Association*. 1981;179(10):1011-1012.

11. Cawley JR, Wright ZM, Meleo K, et al. Concurrent use of rabacfosadine and L‐asparaginase for relapsed or refractory multicentric lymphoma in dogs. *Journal of Veterinary Internal Medicine*. 2020;34(2):882-889. doi:10.1111/jvim.15723

12. Chun R, Garrett LD, Vail DM. Evaluation of a High‐Dose Chemotherapy Protocol with No Maintenance Therapy for Dogs with Lymphoma. *Journal of Veterinary Internal Medicine*. 2000;14(2):120-124. doi:10.1111/j.1939-1676.2000.tb02224.x

13. Davies O, Szladovits B, Polton G, Garden OA, Leo C, Lara‐Garcia A. Prognostic significance of clinical presentation, induction and rescue treatment in 42 cases of canine centroblastic diffuse large B‐cell multicentric lymphoma in the United Kingdom. *Veterinary and Comparative Oncology*. 2018;16(2):276-287. doi:10.1111/vco.12378

14. De Clercq E. Tanovea® for the treatment of lymphoma in dogs. *Biochemical Pharmacology*. 2018;154:265-269. doi:10.1016/j.bcp.2018.05.010

15. Dékay V, Karai E, Füredi A, Szebényi K, Szakács G, Vajdovich P. P-Glycoprotein Activity at Diagnosis Does Not Predict Therapy Outcome and Survival in Canine B-Cell Lymphoma. *Cancers*. 2022;14(16):3919. doi:10.3390/cancers14163919

16. Dervisis NG, Dominguez PA, Sarbu L, et al. Efficacy of temozolomide or dacarbazine in combination with an anthracycline for rescue chemotherapy in dogs with lymphoma. *Journal of the American Veterinary Medical Association*. 2007;231(4):563-569. doi:10.2460/javma.231.4.563

17. Duckett ME, Curran KM, Bracha S, Leeper HJ. Retrospective Evaluation of Melphalan, Vincristine, and Cytarabine Chemotherapy for the Treatment of Relapsed Canine Lymphoma. *Journal of the American Animal Hospitals Association*. 2024;60(1):7-14. doi:10.5326/JAAHA-MS-7372

18. Elliott JW, Cripps P, Marrington AM, Grant IA, Blackwood L. Epirubicin as part of a multi‐agent chemotherapy protocol for canine lymphoma. *Veterinary and Comparative Oncology*. 2013;11(3):185-198. doi:10.1111/j.1476-5829.2011.00311.x

19. Fahey CE, Milner RJ, Barabas K, et al. Evaluation of the University of Florida lomustine, vincristine, procarbazine, and prednisone chemotherapy protocol for the treatment of relapsed lymphoma in dogs: 33 cases (2003–2009). *Journal of the American Veterinary Medical Association*. 2011;239(2):209-215. doi:10.2460/javma.239.2.209

20. Flory AB, Rassnick KM, Al‐Sarraf R, et al. Combination of CCNU and DTIC Chemotherapy for Treatment of Resistant Lymphoma in Dogs. *Journal of Veterinary Internal Medicine*. 2008;22(1):164-171. doi:10.1111/j.1939-1676.2007.0005.x

21. Gardner HL, Rippy SB, Bear MD, et al. Phase I/II evaluation of RV1001, a novel PI3Kδ inhibitor, in spontaneous canine lymphoma. Thamm DH, ed. *PLOS One*. 2018;13(4):e0195357. doi:10.1371/journal.pone.0195357

22. Gillem J, Giuffrida M, Krick E. Efficacy and toxicity of carboplatin and cytarabine chemotherapy for dogs with relapsed or refractory lymphoma (2000–2013). *Veterinary and Comparative Oncology*. 2017;15(2):400-410. doi:10.1111/vco.12176

23. Griessmayr PC, Payne SE, Winter JE, Barber LG, Shofer FS. Dacarbazine as Single‐Agent Therapy for Relapsed Lymphoma in Dogs. *Journal of Veterinary Internal Medicine*. 2009;23(6):1227-1231. doi:10.1111/j.1939-1676.2009.0376.x

24. Hohenhaus AE, Matus RE. Etoposide (VP‐16): Retrospective Analysis of Treatment in 13 Dogs With Lymphoma. *Journal of Veterinary Internal Medicine*. 1990;4(5):239-241. doi:10.1111/j.1939-1676.1990.tb03115.x

25. Hosoya K, Kisseberth WC, Lord LK, et al. Comparison of COAP and UW‐19 Protocols for Dogs with Multicentric Lymphoma. *Journal of Veterinary Internal Medicine*. 2007;21(6):1355-1363. doi:10.1111/j.1939-1676.2007.tb01959.x

26. Intile JL, Rassnick KM, Al-Sarraf R, Chretin JD. Evaluation of the Tolerability of Combination Chemotherapy with Mitoxantrone and Dacarbazine in Dogs with Lymphoma. *Journal of the American Animal Hospital Association*. 2019;55(2):101-109. doi:10.5326/JAAHA-MS-6878

27. Jeong SY. Evaluation of factors influencing survival time in 77 dogs with lymphoma. *Open Veterinary Journal*. 2023;13(9):1124-1134. doi:10.5455/OVJ.2023.v13.i9.8

28. Kaiser CI, Fidel JL, Roos M, Kaser-Hotz B. Reevaluation of the University of Wisconsin 2-Year Protocol for Treating Canine Lymphosarcoma. *Journal of the American Animal Hospital Association*. 2007;43(2):85-92. doi:10.5326/0430085

29. Lautscham EM, Kessler M, Ernst T, Willimzig L, Neiger R. Comparison of a CHOP‐LAsp‐based protocol with and without maintenance for canine multicentric lymphoma. *Veterinary Record*. 2017;180(12):303-303. doi:10.1136/vr.104077

30. LeBlanc AK, Mauldin GE, Milner RJ, LaDue TA, Mauldin GN, Bartges JW. Efficacy and toxicity of BOPP and LOPP chemotherapy for the treatment of relapsed canine lymphoma*. *Veterinary and Comparative Oncology*. 2006;4(1):21-32. doi:10.1111/j.1476-5810.2006.00088.x

31. London CA, Bernabe LF, Barnard S, et al. Preclinical Evaluation of the Novel, Orally Bioavailable Selective Inhibitor of Nuclear Export (SINE) KPT-335 in Spontaneous Canine Cancer: Results of a Phase I Study. Richards KL, ed. *PLOS One*. 2014;9(2):e87585. doi:10.1371/journal.pone.0087585

32. Lucroy MD, Phillips BS, Kraegel SA, Simonson ER, Madewell BR. Evaluation of Single‐Agent Mitoxantrone as Chemotherapy for Relapsing Canine Lymphoma. *Journal of Veterinary Internal Medicine*. 1998;12(5):325-329. doi:10.1111/j.1939-1676.1998.tb02130.x

33. Macewen EG, Rosenthal R, Matus R, Viau AT, Abuchowski A. A preliminary study on the evaluation of asparaginase. Polyethylene glycol conjugate against canine malignant lymphoma. *Cancer*. 1987;59(12):2011-2015. doi:10.1002/1097-0142(19870615)59:12%3C2011::AID-CNCR2820591207%3E3.0.CO;2-M

34. Mastromauro ML, Suter SE, Hauck ML, Hess PR. Oral melphalan for the treatment of relapsed canine lymphoma. *Veterinary and Comparative Oncology*. 2018;16(1):E123-E129. doi:10.1111/vco.12356

35. Meier V, Geigy C, Grosse N, McSheehy P, Rohrer Bley C. Use of Epothilone B (Patupilone) in Refractory Lymphoma and Advanced Solid Tumors in Dogs. *Journal of Veterinary Internal Medicine*. 2013;27(1):120-125. doi:10.1111/jvim.12019

36. Mellanby RJ, Herrtage ME, Dobson JM. Treatment of canine lymphoma by veterinarians in first opinion practice in England. *Journal of Small Animal Practice*. 2002;43(5):198-202. doi:10.1111/j.1748-5827.2002.tb00056.x

37. Moore AS, Imondi AR, De Souza PL, Wood CA. Intravenous administration of 9‐aminocamptothecin to dogs with lymphoma. *Veterinary and Comparative Oncology*. 2003;1(2):86-93. doi:10.1046/j.1476-5829.2003.00012.x

38. Moore AS, Ogilvie GK, Vail DM. Actinomycin D for reinduction of remission in dogs with resistant lymphoma. *Journal of Veterinary Internal Medicine*. 1994;8(5):343-344. doi:10.1111/j.1939-1676.1994.tb03247.x

39. Moore AS, Cotter SM, Rand WM, et al. Evaluation of a Discontinuous Treatment Protocol (VELCAP‐S) for Canine Lymphoma. *Journal of* *Journal of Veterinary Internal Medicine*. 2001;15(4):348-354. doi:10.1111/j.1939-1676.2001.tb02328.x

40. Moore AS, London CA, Wood CA, et al. Lomustine (CCNU) for the Treatment of Resistant Lymphoma in Dogs. *Journal of* *Veterinary Internal Medicine*. 1999;13(5):395-398. doi:10.1111/j.1939-1676.1999.tb01452.x

41. Northrup NC, Gieger TL, Kosarek CE, et al. Mechlorethamine, procarbazine and prednisone for the treatment of resistant lymphoma in dogs. *Veterinary and Comparative Oncology*. 2009;7(1):38-45. doi:10.1111/j.1476-5829.2008.00170.x

42. O’Connell K, Thomson M, Morgan E, Henning J. Procarbazine, prednisolone and cyclophosphamide oral combination chemotherapy protocol for canine lymphoma. *Veterinary and Comparative Oncology*. 2022;20(3):613-622. doi:10.1111/vco.12814

43. Parker AS, Burton JH, Curran KM, Wolf-Ringwall A, Thamm DH. Early progression during or after cyclophosphamide, doxorubicin, vincristine, and prednisone chemotherapy indicates poor outcome with rescue protocols in dogs with multicentric lymphoma. *Journal of Veterinary Internal Medicine*. 2024;38(4):2282-2292. doi:10.1111/jvim.17139

44. Parsons-Doherty M, Poirier VJ, Monteith G. The efficacy and adverse event profile of dexamethasone, melphalan, actinomycin D, and cytosine arabinoside (DMAC) chemotherapy in relapsed canine lymphoma. *Canadian Veterinary Journal* 2014;55(2):175-80

45. Rassnick KM, Mauldin GE, Al‐Sarraf R, Mauldin GN, Moore AS, Mooney SC. MOPP Chemotherapy for Treatment of Resistant Lymphoma in Dogs: A Retrospective Study of 117 Cases (1989–2000). *Journal of Veterinary Internal Medicine*. 2002;16(5):576-580. doi:10.1111/j.1939-1676.2002.tb02390.x

46. Saba CF, Hafeman SD, Vail DM, Thamm DH. Combination chemotherapy with continuous L-asparaginase, lomustine, and prednisone for relapsed canine lymphoma. *Journal of Veterinary Internal Medicine*. 2009;23(5):1058-1063. doi:10.1111/j.1939-1676.2009.0357.x

47. Saba CF, Vickery KR, Clifford CA, et al. Rabacfosadine for relapsed canine B‐cell lymphoma: Efficacy and adverse event profiles of 2 different doses. *Veterinary and Comparative Oncology*. 2018;16(1). doi:10.1111/vco.12337

48. Saba CF, Thamm DH, Vail DM. Combination Chemotherapy with L‐Asparaginase, Lomustine, and Prednisone for Relapsed or Refractory Canine Lymphoma. *Journal of Veterinary Internal Medicine*. 2007;21(1):127-132. doi:10.1111/j.1939-1676.2007.tb02938.x

49. Sadowski AR, Gardner HL, Borgatti A, et al. Phase II study of the oral selective inhibitor of nuclear export (SINE) KPT-335 (verdinexor) in dogs with lymphoma. *BMC Veterinary Research*. 2018;14(1):250. doi:10.1186/s12917-018-1587-9

50. Silver M, Rusk A, Phillips B, et al. Evaluation of the Oral Antimitotic Agent ( ABT ‐751) in Dogs with Lymphoma. *Journal of Veterinary Internal Medicine*. 2012;26(2):349-354. doi:10.1111/j.1939-1676.2012.00892.x

51. Sirivisoot S, Teewasutrakul P, Rungsipipat A, Tangkawattana S, Techangamsuwan S. Transcriptome analysis of *ABCB1* , *ABCG2* and the *BCL2/BAX* ratio in refractory and relapsed canine lymphomas under treatment and rescue protocol. *Acta Veterinaria*. 2018;68(1):16-31. doi:10.2478/acve-2018-0002

52. Smallwood K, Tanis J, Grant IA, et al. Evaluation of a multi‐agent chemotherapy protocol combining dexamethasone, melphalan, actinomycin D, and cytarabine for the treatment of resistant canine non‐Hodgkin high‐grade lymphomas: a single centre’s experience. *Veterinary and Comparative Oncology*. 2019;17(2):165-173. doi:10.1111/vco.12457

53. Smith AA, Lejeune A, Kow K, Milner RJ, Souza CHM. Clinical Response and Adverse Event Profile of Bleomycin Chemotherapy for Canine Multicentric Lymphoma. *Journal of the American Animal Hospital Association*. 2017;53(2):128-134. doi:10.5326/JAAHA-MS-6598

54. Tanis J ‐B., Mason SL, Maddox TW, et al. Evaluation of a multi‐agent chemotherapy protocol combining lomustine, procarbazine and prednisolone (LPP) for the treatment of relapsed canine non‐Hodgkin high‐grade lymphomas. *Veterinary and Comparative Oncology*. 2018;16(3):361-369. doi:10.1111/vco.12387

55. Teske E, Rutteman GR, Van Heerde P, Misdorp W. Polyethylene glycol-L-asparaginase versus native L-asparaginase in canine non-Hodgkin’s lymphoma. *European Journal of Cancer and Clinical Oncology*. 1990;26(8):891-895. doi:10.1016/0277-5379(90)90193-W

56. Thamm DH, Joseph JK, Rose BJ, Meuten TK, Weishaar KM. Phase-I trial of survivin inhibition with EZN-3042 in dogs with spontaneous lymphoma. *BMC Veterinary Research*. 2020;16(1):97. doi:10.1186/s12917-020-02317-3

57. Treggiari E, Elliott JW, Baines SJ, Blackwood L. Temozolomide alone or in combination with doxorubicin as a rescue agent in 37 cases of canine multicentric lymphoma. *Veterinary and Comparative Oncology*. 2018;16(2):194-201. doi:10.1111/vco.12335

58. Troedson K, Ignatenko N, Fejos C, Zablotski Y, Hirschberger J. Maintenance treatment in relapsed canine lymphoma after a short L-CHOP protocol *Tierärztliche Praxis Kleintiere*.*Heimtiere* 2021;49(03):185-194

59. Turner AI, Hahn KA, Rusk A, et al. Single Agent Gemcitabine Chemotherapy in Dogs with Spontaneously Occurring Lymphoma. *Journal of Veterinary Internal Medicine*. 2006;20(6):1384-1388. doi:10.1111/j.1939-1676.2006.tb00755.x

60. Vail DM, Thamm DH, Reiser H, et al. Assessment of GS-9219 in a Pet Dog Model of Non-Hodgkin’s Lymphoma. *Clinical Cancer Research*. 2009;15(10):3503-3510. doi:10.1158/1078-0432.CCR-08-3113

61. Van Vechten M, Helfand SC, Jeglum KA. Treatment of Relapsed Canine Lymphoma With Doxorubicin and Dacarbazine. *Journal of Veterinary Internal Medicine*. 1990;4(4):187-191. doi:10.1111/j.1939-1676.1990.tb00896.x

62. Weishaar KM, Wright ZM, Rosenberg MP, et al. Multicenter, randomized, double‐blinded, placebo‐controlled study of rabacfosadine in dogs with lymphoma. *Journal of Veterinary Internal Medicine*. 2022;36(1):215-226. doi:10.1111/jvim.16341

63. Yamazaki H, Miura N, Lai YC, et al. Effects of toceranib phosphate (Palladia) monotherapy on multidrug resistant lymphoma in dogs. *The Journal of Veterinary Medical Science*. 2017;79(7):1225-1229. doi:10.1292/jvms.16-0457

64. Zandvliet M, Rutteman GR, Teske E. Prednisolone inclusion in a first-line multidrug cytostatic protocol for the treatment of canine lymphoma does not affect therapy results. *The Veterinary Journal*. 2013;197(3):656-661. doi:10.1016/j.tvjl.2013.04.022

65. Zimmerman K, Walsh KA, Ferrari JT, Keuler NS, Atherton MJ, Lenz JA. Evaluation of mechlorethamine, vinblastine, procarbazine, and prednisone for the treatment of resistant multicentric canine lymphoma. *Veterinary and Comparative Oncology*. 2023;21(3):503-508. doi:10.1111/vco.12913
